# The use of technology in higher education teaching by academics during the COVID-19 emergency remote teaching period: a systematic review

**DOI:** 10.1186/s41239-022-00364-4

**Published:** 2022-12-14

**Authors:** McQueen Sum, Alis Oancea

**Affiliations:** Department of Education, University of Oxford, 15 Norham Gardens, Oxford, OX2 6PY UK

**Keywords:** Systematic review, Higher education, Technology use, Pandemic, Emergency remote teaching

## Abstract

This paper presents a systematic review of scholarly efforts that uniquely emerged at the onset of the COVID-19 pandemic and focused primarily on higher education teachers’ perspectives on technology use and on associated changes in the relationship between teachers and students amidst the transition to emergency remote teaching worldwide. Our narrative synthesis of 32 studies, the majority of which come from lower-and middle-income countries/regions, suggests that numerous factors interact to shape academics’ technology use in emergency remote teaching across higher education contexts. We report strong findings of teachers’ resilience and resourcefulness in their self-exploration of various technologies and teaching strategies in response to the continued severity of the pandemic. Ultimately, this review suggests directions for further research on engaging educational leaders and faculty in reimagining teaching as not only a core academic function of higher education, but also, and importantly, a humanising experience shaped by an ethics of care.

## Review of literature and research questions

Since the continued devastating spread of COVID-19 across continents from early 2020, the coronavirus pandemic has led to massive numbers of hospitalisations and deaths around the world, abruptly upending public health and many other domains of life. As the disaster has unfolded, a multitude of sweeping challenges have continued to reshape the global higher education (‘HE’) landscape. With HE institutions (‘HEIs’) worldwide closing their campuses in Spring 2020, teachers were forced to make a hasty transition from typically in-person teaching configured in physically proximate space to alternative teaching approaches in response to the COVID-19 emergency (Crawford et al., 2020).

The term ‘emergency remote teaching’ (‘ERT’) is used by Hodges et al. (2020) and subsequent literature to denote the rapid and putatively ephemeral shift to remote teaching to continue teaching and learning during emergencies. Although ‘ERT’ and ‘online teaching’ may be two domains with considerable overlaps, ‘online teaching’ is importantly distinguished from ‘ERT’ as it includes teaching and learning arising from a prolonged collective effort in curriculum planning and instructional design from a wide range of stakeholders pre-launching (Hodges et al., 2020).

Despite the growing literature on ERT, few efforts had been made to review this body of research systematically at the time of conducting this review (see Table 1 for a few exceptions). Since there have been abundant discussions on the perspectives of students at the HE level during COVID-19 [see, for example, Chakraborty et al. (2021) on Indian students’ opinions on various aspects of ERT; Mok et al. (2021) on Hong Kong students’ evaluation of their learning experiences during ERT; Resch et al. (2022) on social and academic integration of Austrian students; and Salas-Pilco et al. (2022) for a systematic review focusing on student engagement in Latin American HE], our review focuses systematically on synthesising the body of worldwide literature on teachers’ perspectives on technology use during the period of ERT. Moreover, much attention has been devoted to medical education (Rajab et al., 2020; see also Table 1) and STEM education since the coronavirus outbreak (Amunga, 2021; Bond et al., 2021; Gaur et al., 2020; Singh-Pillay & Naidoo, 2020). Our review focuses on the less explored perspectives of humanities, arts, and social sciences (HASS) teachers—whose perceived difficulties of using digital technologies in teaching were reportedly distinct from those of their counterparts in other disciplines, both before (Mercader & Gairín, 2020) and during the COVID-19 outbreak (Wu et al., 2020).

**Table 1:** Prior systematic reviews published in 2020–2021 pertaining to technology use in higher education teaching in the COVID-19 context

Paper	Review foci	Number of articles reviewed	Range of publication date	Highlights of findings
Bond et al. (2021)	To map the higher education literature conducted on ERT in the early stages of the pandemic outbreak	282	Up to the first week of December 2020	Most studies on ERT focused largely on the perceptions of undergraduate students of teaching and learning in STEMM-related fields
Dedeilia et al. (2020)	To identify COVID-induced challenges and propose innovations of medical and surgical education	61	Up to 18 April 2020	Concerns such as shortage of protective gear and overwhelming stress on medical students are reported. Mitigations including telemedicine and attending to trainee’s mental health are discussed
Gordon et al. (2020)	To describe and evaluate the developments in medical education in response to COVID-19	49	From 1 December 2019 to 24 May 2020	Developments in remote medical education were rapidly deployed as alternatives to clinical placements to support learning during the initial outbreaks of COVID
Na and Jung (2021)	To identify the challenges university instructors faced when teaching online during COVID-19	8	From 1 January 2020 to 30 April 2021	Seven different categories of teachers’ challenges are identified; design opportunities and support needed to overcome these challenges are proposed
Talib et al. (2021)	To evaluate the impact of transitioning to teaching and learning online in the context of COVID-19 on teachers, students, and education as a whole	47	Later than 2019	Numerous opportunities and challenges of teaching and learning during the times of COVID are reported and discussed from the perspectives of various stakeholders

Prior to COVID-19, a respectable amount of scholarly work was devoted to the development and adaptation of theoretical models to identify, explain, and even predict factors that influenced technology use in educational contexts (Granić & Marangunić, 2019). But Lee and Jung (2021) argue that ‘in higher education contexts, crisis-driven changes may happen differently from pre-planned, voluntary change, and that factors influencing crisis-driven changes are different from those influencing voluntary changes; as reported in previous studies based on technology acceptance theories and models’ (p. 16). Given the novelty of COVID-19, few studies have been conducted to explicate the factors shaping HE teachers’ decisions about, and experiences of, technology use in the unique context of the global pandemic [see Mittal et al. (2021) for an exception that studies faculty members in Northern India and Lee and Jung (2021) for another study on South Korean university educators]. Therefore, the first question that this review aims to answer is: *How have different potential factors, as identified by teachers in the included studies, shaped teachers’ technology use across various higher education contexts during the COVID-19 emergency remote teaching period?*

Existing scholarly efforts that aim to provide an overview of the literature focus predominantly on a bifurcated discussion of the opportunities and challenges, or advantages and disadvantages pertinent to using technologies in teaching during the COVID-19 crisis (Adedoyin & Soykan, 2020; Dhawan, 2020; Pokhrel & Chhetri, 2021; Stewart, 2021). We therefore frame the second research question in a way that circumvents a binary pros-and-cons discussion of the implications of technology use in times of the COVID pandemic, as already well-documented in the literature. Hence, our second question is: *What are the implications of technology use in COVID-19 emergency remote teaching from the perspectives of higher education teachers?*

The broader term ‘technology’ (in the singular form) used in the review questions includes the socio-cultural contexts of the educational settings in which technology use is situated. The discussion of ‘context’ is of particular importance (Selwyn, 2022). Although pre-COVID studies (such as Broadbent & Poon, 2015; Liu et al., 2020) offered valuable insights into technology use in HE teaching, the pandemic brought about starkly and often perilously different contexts for research as well as for teaching and learning (Stewart, 2021; Williamson et al., 2020).

We use the term ‘technologies’ in its plural form throughout this review, in a narrower sense, meaning specifically the wide range of digital tools and systems and other technical resources that are used for pedagogical purposes. These can include but are not limited to electronic hardware devices, software systems, online services, and social media. We note, however, that the meanings attached to the term ‘technologies’ may be substantively different across contexts. Some of the studies included in this review, as we will show below, extend it to other-than-digital forms of technologies, leading to results beyond our initial scope of research. As a result, the use of (digital) technologies is understood in this review as an *often necessary but not sufficient* condition for ERT—a novel concept to many teachers who had been using various ‘technologies’ in other ways in facilitating their teaching for years before the COVID-19 outbreak.

## Methodology

Characterised by the principles of replicability and transparency, a systematic review aims to ‘review ... existing research using explicit, accountable rigorous research methods’ (Gough et al., 2017, p. 4). This methodology is used because it helps elucidate the current understanding and available evidence of the above research questions, clarify any replication of existing research findings, and inform future research and policy directions in HE teaching in a systematic and trustworthy manner. Below is a detailed, transparent report of the processes involved in conducting this systematic review.

### Inclusion/exclusion criteria

Our review is restricted to peer-reviewed journal articles that report original empirical studies written in English and/or simplified Chinese. Papers written in these two languages account for a high volume of worldwide literature published at the onset of the COVID-19 outbreak. Also, Chinese studies are particularly valuable for this review, for mainland China was the first region affected by COVID-19 and its HE system was amongst the first to respond to the challenges ensuing from the spread of coronavirus.

Since the review seeks to capture a ‘snapshot’ of perspectives on technology use by teachers during the immediate COVID-19 outbreak, only articles published in 2020 (including those published online ahead of print that year) were eligible for review. Included publications may cover any country/region worldwide but should systematically gather data from teachers other than the authors themselves and focus primarily on the perspectives of HASS teachers on matters pertaining to technology use in ERT in HE settings. Opinion pieces, editorials, reflection articles on one’s own practice, conference papers, and books are not within the purview of this review (see Appendix 1 for detailed inclusion/exclusion criteria).

### Search strategy

Prior to conducting the database search, we piloted and modified the search strings several times. Our final search strategy is a combination of Boolean operators and variations of four key terms: ‘higher education’, ‘technology’, ‘teaching’, and ‘COVID-19’ (see Appendix 2 for detailed search terms).

### Screening and selection

On 13 January 2021, a targeted search returned 4204 records indexed in fourteen databases including Scopus, Web of Science, and three Chinese databases (see Appendix 3 for PRISMA flow diagram and the complete list of databases). From these, we extracted 20 different papers at random to screen by title and abstract independently by applying the inclusion/exclusion criteria, and with the intention to repeat the process until unanimous agreement was reached. Having achieved full inter-reviewer agreement in our first attempt and after a further calibration session, we then proceeded to de-duplication and title-and-abstract screening, after which only 129 papers remained for full-text retrieval and further screening. Meanwhile, 16 relevant publications from various other sources were also identified and passed the initial screening. We then examined the full text of the resulting total of 145 articles and excluded any that did not fulfil the inclusion criteria, leading to a set of 40 studies to be considered for review.

### Quality and relevance assessment and content extraction

To assess the 40 papers’ quality and relevance to this review, we adapted the assessment rubric from Oancea et al. (2021) (see Appendix 4). In parallel with the quality assessment, we developed a grid for content extraction by piloting on three papers, after which multiple revisions of the extraction grid were made. Then both authors used the updated extraction grid (see Appendix 5) and extracted content from two full papers independently to check for inter-reviewer agreement. In subsequent communications, discrepancies of our extraction were reconciled and the final quality thresholds for inclusion were agreed upon. As of May 2021, after excluding 8 papers of low quality, the final corpus for review comprised 32 articles.

### Analysis and synthesis

We developed an initial coding scheme with broad theme boundaries based on the research questions, and resolved any conflicting views. We coded line-by-line the extracted data both deductively and inductively: we first applied the pre-configured coding scheme to the full set of data, and then updated and re-applied the coding scheme to include further themes identified through inductive coding. For example, we realised that the category of ‘ethical use of technology’ spanned the themes of ‘pedagogical implications’ and ‘work-related implications’. As a result we categorised it under a separate theme titled ‘cross-cutting implications’. After multiple rounds of scheme refinement and iterative coding which started in June 2021, the process of synthesis concluded in late December 2021.

The research synthesis is presented narratively; note that we integrated quantitative findings (for example, from surveys) descriptively into the narrative analysis, as in most cases the samples were not representative, the analysis was largely descriptive and findings from qualitative answers to open questions were presented in detail.

### Limitations

Our review did not include insights from reflection pieces (such as Czerniewicz et al., 2020; Jandrić et al., 2020; Joseph & Trinick, 2021) and reports not published in peer-reviewed journals (such as Ferdig et al., 2020); these exclusions are not a judgment on either the quality or the level of insight of such pieces, nor on the modes of research and scholarship that they embody. This decision, as well as the focus on studies published in English and Chinese, limit the extent to which this review covers the experiences of ERT technology use by teacher populations across the world.

Due to our international remit, another limitation is the integration of findings grounded in different local contexts and HE environments. We overcome this partially by extracting from each paper the context in which teachers’ technology use is situated and taking such information into account when narratively integrating data across studies and presenting our review findings (see Appendix 5). However, the inconsistent terminology used to allude to the notions of ‘technology’ and ‘emergency remote teaching’ in the reviewed articles poses a major challenge to our cross-context comparison [see discussion on the jingle-jangle fallacy in Sum and Oancea (2021)]. Another review conducted by Bond et al. (2021) also found at least ten different terms used for ‘online teaching’ (including ‘emergency remote teaching’) in their selected papers.

Although uniformly agreed-upon definitions of these terms are absent (Singh & Thurman, 2019), the nuances of concepts underlying them have not been given due consideration in the majority of the studies reviewed (see “Description of included articles” section). Further terminological complexity arises from the imperfect overlap between Chinese and English vocabularies. Whilst we tried to overcome this by extracting information on each study’s conceptualisation of ‘technology’ and ‘ERT’ (see Appendix 5) and accompanying translations with original Chinese terms (for example, the phrase ‘线上教学’ in Chinese can be sometimes translated into ‘online teaching and learning’), we acknowledge that terminological and translation gaps remain in our cross-context synthesis of the selected literature.

## Findings

### Description of included articles

Included in our final synthesis are 32 empirical research studies covering 71 countries and reporting perspectives from 4725 HE teachers altogether. Of these, the largest proportion focuses on the HE context in Asia (n = 15), followed by Europe (n = 7) and Africa (n = 6) (see Table 2). Given our inclusion of articles indexed in Chinese databases, Mainland China alone is the focal context of n = 5 studies. A wide range of subject areas in HASS disciplines are covered (see Table 3). Studies using qualitative data are most common (n = 14) (see Table 4), and a sample size of fewer than 50 teachers is often reported (n = 21) (see Table 5). Appendix 6 presents a summary of the characteristics of included studies.

**Table 2:** Geographical distribution of the included studies (N = 32)

Africa	N	Americas	N	Asia	N	Europe	N	Oceania	N	Other	N
Algeria	1	Ecuador, Italy, and Spain	1	Bangladesh	1	Ecuador, Italy, and Spain	0^a^	Australia	1	Global	1
Egypt	1	Trinidad and Tobago	1	India	1	Spain	1				
Ghana	2	United States	1	Indonesia	1	Turkey	1				
South Africa	2			Israel	1	United Kingdom	3				
				Lebanon	1	13 European countries	1				
				Mainland China	5						
				Pakistan	1						
				Saudi Arabia	2						
				South Korea	1						
				The Philippines	1						
Sub-total	6		3		15		6		1		1

^a^To avoid double counting, this paper has only been counted once, under ‘Americas’

**Table 3:** Disciplinary areas in the included articles

Disciplines	Number of studies	% of 32 studies
Multi-discipline (primarily in social sciences and humanities)	10	31
Education	8	25
Language studies	6	19
Business and economics	2	6
Translation/interpretation studies	2	6
Other	4	13
Total	32	100

**Table 4:** Research approach of included articles

Approach	Number of studies	% of 32 studies
Predominantly qualitative	14	44
Predominantly quantitative	10	31
Mixed methods	8	25
Total	32	100

**Table 5:** Higher education teacher sample size of the included articles

Sample size of higher education teachers	Number of studies	% of 32 studies
1 to 9	6	19
10 to 49	15	47
50 to 99	2	6
100 to 499	6	19
500 to 999	2	6
1000 or more	1	3
Total	32	100

Exactly half of the studies (n = 16) have a local remit (see Table 6), amongst which many recruited fellow academics from the authors’ institutions (n = 14). As noted by several researchers in their papers, the public health emergency and its concomitant restrictions had in various ways altered the methods for research and data collection, including shifting to a local focus whilst access to other settings was limited.

**Table 6:** Remit of included articles

Remit	Number of studies	% of 32 studies
Local	16	50
Provincial	5	16
National	8	25
Regional	2	6
Global	1	3
Total	32	100

Authors of three quarters of the reviewed studies (n = 24) obtained data from participants remotely, either by phone or online. Much empirical data were collected in a space that was relatively new and unfamiliar to the researcher and the researched during a time when both individuals were coping with not only the expected expeditious embrace of various technologies for ERT but also, amongst other things, the physical and psychological burden posed by the coronavirus pandemic. Hence, this review integrates, in a systematic and holistic fashion, data from the discrete, often inevitably limited, yet valiant research initiatives undertaken in different countries during the periods of drastic increases in infections and deaths at the incipient phase of the COVID-19 outbreak.

In terms of substantive focus, whilst most of the included studies describe ‘what’ and/or ‘how’ technologies were being used by teachers during ERT (n = 14) and offer a dichotomous pros-or-cons narrative of technology use for ERT (n = 21), often vis-à-vis in-person teaching prior to COVID-19, some (n = 7) also examine the wider implications for teachers and HE at large.

Due partly to the novelty of COVID-19 and the haste with which research was conducted, the conceptualisation of technology and its relation with remote teaching in times of COVID-19 is either weak or largely absent in the majority of the reviewed studies. Technologically deterministic views seem prevalent in the literature reviewed. Many studies place ‘technology’ as the centre of inquiry and underscore the palpable ‘impact’ that various technical objects impose on teaching. For example, the attribution of recent pedagogical innovations and educational developments to technological advancements features prominently in the introductory paragraphs of numerous papers. Some assert that the emergence of social networking sites has begun to direct all walks of life including the ways in which teaching has been carried out since before the pandemic. Additionally, the discussion of ‘technology-enabled’ and ‘technology-enhanced’ teaching used in some articles implies that ‘technology’ plays an almost indispensable role in teaching and that teaching would be seriously disrupted without it. In contrast, there was little awareness in many of these papers of the extent to which technologies may carry political or commercial agendas or may be underpinned by complex ideologies and social structures (Selwyn et al., 2020). This echoes the conclusions of pre-COVID research by An and Oliver (2021) and Costa et al. (2019) that theoretical understanding of ‘technology’ in educational research is under-developed.

### A brief narrative of ERT experiences from teachers’ perspectives

An eclectic range of technological artefacts and their uses during ERT across HE settings is reported in the studies. Cases of initial technology use range widely from straightforward approaches such as uploading teaching materials online to (mis)uses such as creating excessive recorded lectures and assignments. What is common, however, across reports in most studies is the acutely negative sentiments of intimidation, angst, confusion, and even despair of ERT amongst teachers at the outset of the transitioning period. It gave teachers great shock and pain to make a forced, often slapdash migration to ERT—a terrain that many of them were unfamiliar with and uncertain of—whilst juggling with their home and other work responsibilities during the distressing period. In addition to the psychological burden, teachers were worried about the well-being of their students, particularly those from underprivileged backgrounds and in vulnerable environments. Across HE settings worldwide, teachers had on average less than a week’s preparation time, leaving them feeling woefully unprepared. Hence, it is unsurprising that the majority of teachers in the studies reviewed found the immediate phase of migration to ERT burdensome and emotionally exhausting. Yet, some sought a silver lining and considered ERT as a creative challenge and an opportunity for a long-needed meaningful reflection and overhaul of HE teaching practices.

We mapped each included article’s findings about teachers’ overall attitudes towards ERT using the World Bank’s classification of country development (2020) (see Table 7). For studies not examining teachers’ attitudes directly, we inferred negative attitudes from teachers’ reports of dissatisfaction and frustrations over the challenges in ERT, and any indication of concern and anxiety; positive attitudes were inferred from teachers’ expressions of satisfaction and awareness of benefits brought by ERT, and any indication of optimism and hope.

**Table 7:** Teachers’ overall attitudes towards emergency remote teaching (ERT) and its concomitant technology use as implied in the articles reviewed (categorised based on World Bank (2020)’s country classification by income)

References	Context(s) of focus	Teacher participants’ overall attitudes towards ERT and its concomitant technology use^a^				
		Mostly negative	More negative	Mixed response	More positive	Mostly positive
Studies focussing on high-income countries/regions (11)						
Marshalsey and Sclater (2020)	Australia			x		
Hadar et al. (2021)	Israel				x	
Alqabbani et al. (2020)	Saudi Arabia					x
Alsadoon and Turkestani (2020)	Saudi Arabia	x				
Bailey and Lee (2020)	South Korea			x		
Sales et al. (2020)	Spain				x	
Mideros (2020)	Trinidad and Tobago				x	
Eringfeld (2021)	United Kingdom			x		
Kidd and Murray (2020)	United Kingdom				x	
Watermeyer et al. (2021)	United Kingdom		x			
Cutri et al. (2020)	United States			x		
Studies focussing on upper-middle-income countries/regions (10)						
Gao and Zhang (2020)	China (Mainland)			x		
Lu (2020)	China (Mainland)				x	
Ren (2020)	China (Mainland)				x	
Tang et al. (2020)	China (Mainland)				x	
Zeng (2020)	China (Mainland)				x	
Diningrat et al. (2020)	Indonesia	x				
Mouchantaf (2020)	Lebanon			x		
Khoza and Mpungose (2020)	South Africa			x		
Tanga et al. (2020)	South Africa	x				
Akyürek (2020)	Turkey		x			
Studies focussing on lower-middle-income countries/regions (8)						
Ghounane (2020)	Algeria		x			
Khan et al. (2020)	Bangladesh	x				
Sobaih et al. (2020)	Egypt		x			
Dampson et al. (2020)	Ghana		x			
Gyampoh et al. (2020)	Ghana			x		
Joshi et al. (2020)	India	x				
Said et al. (2021)	Pakistan		x			
Callo and Yazon (2020)	Philippines		x			
Studies focussing on multiple countries/regions (3)						
Scherer et al. (2021)	58 countries globally			x		
Tartavulea et al., 2020	13 European countries			x		
Tejedor et al. (2020)	Spain, Italy, and Ecuador			x		
Total		5	7	11	8	1
Percentage of total		16%	22%	34%	25%	3%

^a^We categorise teachers’ attitudes as reported by each paper into five categories (namely ‘mostly negative’, ‘more negative’, ‘mixed response’, ‘more positive’, and ‘mostly positive’) by weighing the strength of evidence for both positive and negative attitudes of teachers reported in and/or inferred from each included study. For example, for teachers’ attitudes to be categorised as ‘mostly negative’, the paper has to (1) present strong evidence for negative attitudes from teachers’ reports of dissatisfaction and frustrations over the challenges in ERT, and any indication of concern and anxiety, and (2) present little or no evidence for positive attitudes which can be inferred from teachers’ expressions of satisfaction and awareness of benefits brought by ERT, and any indication of optimism and hope

Reports by teachers from higher-income countries/regions were more positive whilst those from lower-and middle-income countries/regions tended to be more negative, though with a few exceptions (for example, teachers in mainland China had relatively positive emotional responses and teachers of hearing-impaired students in high-income Saudi Arabia reported overwhelmingly negative emotional responses during the ERT period). In propitious circumstances, teachers’ emotional responses could change substantially over time from apprehension, frustration, and pessimism to relief, affirmation, and an eventual sense of achievement. Sometimes, as teachers gradually became conversant with various technological artefacts and encountered a suitable way of teaching, either serendipitously or after multiple experimentation, they eventually saw ERT as a humbling and rewarding experience. Some teachers evaluated the pedagogical revisions they made during ERT positively and even expressed the intention to keep part of their teaching online or expected to continue to use the technologies employed for ERT in the future.

### Factors shaping technology use by teachers in ERT across HE contexts

The 32 papers reviewed include results on qualitative and quantitative factors identified by teacher participants that potentially shape teachers’ technology use in ERT. Note that these are not always empirically validated, nor explicitly identified as ‘factors’ in the included articles (particularly in qualitative accounts they may be described as reasons, drivers, challenges, barriers, and conditions). Thus, we adopted an open and inclusive definition of factors based on the implied or explicit direction of influence on ERT, and we grouped them thematically. Summary accounts of these thematic groupings based on the data presented in the review corpus are discussed below in descending order of the respective strength of evidence in the reviewed studies (see full references in Table 8).

**Table 8:** Thematic groupings of identified potential factors shaping higher education teachers’ technology use in COVID-19 emergency remote teaching implied in the reviewed studies

Theme	Factor	Details	References
Social-technological	Technical issues surrounding technology use	• Unreliable internet connection	Akyürek (2020), Alsadoon and Turkestani (2020), Callo and Yazon (2020), Diningrat et al. (2020), Gao and Zhang (2020), Gyampoh et al. (2020), Joshi et al. (2020), Zeng (2020)
		• Lack of devices and equipment	Callo and Yazon (2020), Dampson et al. (2020), Gyampoh et al. (2020), Joshi et al. (2020), Khan et al. (2020), Zeng (2020)
		• Inadequacies in infrastructural provision	Akyürek (2020), Mouchantaf (2020), Said et al. (2021), Tartavulea et al. (2020)
	Equity and access in the wider socio-economic context	• Power outage	Dampson et al. (2020), Khan et al. (2020), Said et al. (2021)
		• Long commute for internet	Dampson et al. (2020), Tanga et al. (2020)
		• Financial conditions/affordability, responsibilities, and environment at home	Callo and Yazon (2020), Khoza and Mpungose (2020), Mideros (2020), Tanga et al. (2020)
Institutional	Institutional policies	• Mandatory shift to ERT	Alqabbani et al. (2020), Khoza and Mpungose (2020), Scherer et al. (2021), Tang et al. (2020)
		• Policies and guidelines regulating technology use in teaching	Cutri et al. (2020), Gao and Zhang (2020), Ghounane (2020), Gyampoh et al. (2020), Joshi et al. (2020), Khoza and Mpungose (2020), Marshalsey and Sclater (2020), Sobaih et al. (2020), Watermeyer et al. (2021)
	Institutional support	• Availability of institutional infrastructure	Akyürek (2020), Alqabbani et al. (2020), Marshalsey and Sclater (2020), Mouchantaf (2020), Sobaih et al. (2020)
		• Training provision for teachers and/or students	Alqabbani et al. (2020), Callo and Yazon (2020), Dampson et al. (2020), Mouchantaf (2020), Marshalsey and Sclater (2020), Sobaih et al. (2020), Tanga et al. (2020)
		• Supply of technical support and assistance	Dampson et al. (2020), Gyampoh et al. (2020), Sales et al. (2020), Tang et al. (2020), Watermeyer et al. (2021)
		• Recognition of teachers’ efforts	Joshi et al. (2020)
Individual	Resilience and agency of teachers	• Motivation and commitment to advancing teaching practices	Bailey and Lee (2020), Ghounane (2020), Kidd and Murray (2020), Said et al. (2021), Sales et al. (2020), Tang et al. (2020)
		• Agility, adaptability, and tolerance of uncertainties	Bailey and Lee (2020), Cutri et al. (2020), Hadar et al. (2021), Khoza and Mpungose (2020)
		• Active agency in seeking solutions and innovating technology use in ERT	Akyürek (2020), Gao and Zhang (2020), Hadar et al. (2021), Sales et al. (2020), Said et al. (2021), Sobaih et al. (2020)
	Teachers’ readiness for ERT	• Perceived confidence in enacting ERT	Gyampoh et al. (2020), Khan et al. (2020), Scherer et al. (2021)
		• Perceived preparedness	Alqabbani et al. (2020), Tanga et al. (2020), Watermeyer et al. (2021)
		• Prior experience in ‘online teaching’	Bailey and Lee (2020), Cutri et al. (2020), Khan et al. (2020), Scherer et al. (2021), Tang et al. (2020), Tartavulea et al. (2020), Zeng (2020)
		• Prior experience in using technologies	Alqabbani et al. (2020), Ghounane (2020), Gyampoh et al. (2020), Hadar et al. (2021), Khoza and Mpungose (2020), Marshalsey and Sclater (2020), Mideros (2020), Mouchantaf (2020), Sales et al. (2020)
Pedagogical	Student-centred pedagogies	• Interactivity and student engagement and participation	Akyürek (2020), Bailey and Lee (2020), Dampson et al. (2020), Khan et al. (2020), Kidd and Murray (2020), Marshalsey and Sclater (2020), Mideros (2020), Said et al. (2021), Sales et al. (2020), Tang et al. (2020), Zeng (2020)
		• Consideration of different students’ needs and well-being during ERT	Alsadoon and Turkestani (2020), Cutri et al. (2020), Hadar et al. (2021), Kidd and Murray (2020), Said et al. (2021)
		• Students’ preference for, and familiarity with, technologies	Ghounane (2020), Sales et al. (2020), Sobaih et al. (2020)
	Teaching beliefs and practices	• Disciplinary differences in teaching beliefs and practices	Gao and Zhang (2020), Hadar et al. (2021), Joshi et al. (2020), Marshalsey and Sclater (2020), Mideros (2020), Ren (2020), Said et al. (2021), Sobaih et al. (2020), Watermeyer et al. (2021)
Peer	Information sharing amongst colleagues	• Mutual exchanges, inspiration, and empowerment in newly formed networking spaces online	Khoza and Mpungose (2020), Ren (2020), Said et al. (2021), Scherer et al. (2021)
		• Reliance on colleagues, especially those who are technology-proficient, as an uncertainty mitigation strategy	Bailey and Lee (2020), Cutri et al. (2020), Khoza and Mpungose (2020), Mouchantaf (2020), Ren (2020)

#### Social-technological factors

Whilst Tartavulea et al. (2020) note that the transition to ERT can be facilitated by having online platforms and facilities, they also found that access to electronic devices and internet connection can be a luxury. Frequently reported technical concerns by teachers include the unreliability of network conditions, lack of devices and equipment, and limitations of digital infrastructure. These issues are not only powerful barriers to technology use in emergency teaching but they also disproportionately affect teachers and students in lower-income countries/regions. Note, however, that even in the context of an affluent country like the United States, teachers and students may report inequitable access to the necessities of ERT from home (Cutri et al., 2020; Sales et al., 2020).

Beneath the surface of these technical difficulties are the imbalanced allocation of resources and entrenched socio-economic problems which most commonly beset lower-and middle-income countries and regions (Tanga et al., 2020). Whilst the issues teachers face are highly contextualised, a considerable number of students come from underprivileged backgrounds. Even before the pandemic hit, these students had been confronting different challenges such as, particularly in lower-income countries, frequent commute of several miles from rural areas to the city for internet connection. Even if internet access were provided at home, these students would still need to overcome problems of intermittent or no power supply in their localities. In addition, during lockdowns they may shoulder more home-care responsibilities, sometimes in overcrowded or even abusive home environments.

Some teachers were also amongst vulnerable groups and had limited access to the internet at home, for example due to the sharing of cellular data with household members, and therefore exposed themselves to greater health risks by visiting commercial establishments such as cafés with free internet provision in order to teach remotely. Compounding this predicament is that HE teachers reported that they often had little information about students’ backgrounds, which hindered their efforts to address students’ educational and psychological needs and any equity issues pertinent to their studies (Cutri et al., 2020). These technical complications are situated in specific social contexts and have been a major hindrance to technology use in ERT.

#### Institutional factors

In most of the studies reviewed, the migration to ERT was described as mandatory, and teachers’ use of certain applications was often resultant from policies imposed by their institutions—whose regulations on teaching could be heavily influenced by government decisions, for example in universities in Mainland China (Tang et al., 2020). To ensure continuity and safety of teaching and learning in times of upheaval and uncertainty, some HEIs exercised greater control over the ways in which technologies were used in teaching, such as mandating the use of certain Learning Management Systems (LMS) in teaching (Khoza & Mpungose, 2020) or prohibiting asynchronous methods of teaching (Cutri et al., 2020). Whilst some teachers felt that their creative freedoms to use different technologies in their teaching were constrained by institutional policies**,** others sought detailed guidance and perceived the lack of clear institutional protocols as a significant barrier to technology use in this emergency (Sobaih et al., 2020).

Aside from policy, different forms of institutional support (such as the provision of digital infrastructure and training for both teachers and students) could also be of value to teachers in ERT, although the level of support felt by teachers could vary by discipline (Watermeyer et al., 2021). However, the value of technical assistance might be undermined when technology specialists were just as confused as teachers about teaching remotely in emergency times (Gyampoh et al., 2020; Tanga et al., 2020). Another gap in institutional support pointed out by some studies is the lack of recognising teachers’ hardship and efforts in teaching in the form of pecuniary (such as support for procurement of equipment) and non-pecuniary rewards (such as teaching awards) (Joshi et al., 2020).

#### Individual factors

Sometimes teachers resisted institutional policies and employed instead other technologies of their own preference. Individual factors therefore play an important role in shaping teachers’ technology use. Despite the challenges posed by the pandemic, some teachers were tolerant of uncertainties, valiantly departing from their previous pedagogical praxis and forging ahead with ‘pedagogical agility’ (Kidd & Murray, 2020)—the flexibility of adapting to the new teaching conditions in rapid yet meaningful ways. Resilient and adaptive, these teachers ‘rolled up their sleeves’ and worked around the clock to seek teaching solutions and countermeasures through constant, active self-exploration (Sales et al., 2020). Some music teachers, for instance, would make immediate remedies for the connection disruptions to synchronous lessons by providing students with recordings of their playing as examples (Akyürek, 2020). In an Israeli college, teacher educators incorporated topics like ‘distance learning’ into the teacher training curriculum to reflect the new circumstances of teaching (Hadar et al., 2021). One teacher educator even painted a wall at home with special paint to make it into a ‘blackboard’ where his writings were presented and screened to students (Hadar et al., 2021). These are just a few of the many manifestations of teachers’ agentic creativity and ongoing inventiveness in innovating their own use of technologies and resources despite the presence of severe constraints in ERT times.

In terms of readiness, despite receiving considerable institutional support in some cases, teachers often felt ill-prepared for ERT and doubtful of their abilities in using various technologies to teach (Scherer et al., 2021), and only a minority felt rather ready for ERT (Alqabbani et al., 2020). The studies reviewed discussed the variation in teachers’ readiness for ERT in relation to gender, academic discipline, and country context (Scherer et al., 2021). For example, in predominantly high-income economies teachers moved from a customary integration of technologies in pre-COVID teaching to fully-online ERT (Mideros, 2020; Sales et al., 2020). But not all teachers and students had had the opportunities to familiarise themselves with various technologies (including otherwise widely used applications like Word processing) prior to COVID-19 (Gyampoh et al., 2020). Whilst experienced online teachers felt more prepared and expected themselves to employ more frequently a wide array of technologies in teaching, across HE contexts many teachers had seriously limited prior experience in ‘online teaching’ and were apprehensive about using technologies for teaching purposes (Bailey & Lee, 2020). Besides, being experienced in ‘online teaching’ does not necessarily translate to successful handling of ERT, given the limited time frame and the stressful and even traumatising circumstances at the outset of the crisis.

#### Pedagogical factors

Across HE settings, teachers considered how to connect and engage dislocated groups of students through technologies, how to empower students to explore beyond the curriculum as students gained more control over what and how they study in the shifting context of teaching and learning (Mideros, 2020), and how to reconfigure spaces in ways that provide students with a nourishing, inter-connected intellectual environment despite being physically apart during the ERT period (Kidd & Murray, 2020). In Australia, teachers were especially concerned about first-year students, as the southern hemisphere’s Autumn 2020 was their very first term at the university. In addition to providing students with considered feedback, these teachers employed strategies such as the online polls and hand-raising functions on various EdTech platforms (Zeng, 2020), or made students the host of Blackboard Collaborate in order for teaching to be more engaging (Marshalsey & Sclater, 2020).

As coronavirus infections spread, teachers also attended to students’ emotional and educational well-being. Some teacher educators in the United Kingdom offered one-on-one tutorials online to establish personal connections with student teachers and monitor their progress (Kidd & Murray, 2020). A teacher in Pakistan went the extra mile to care for the students living in far-flung areas without internet access by sending them CD recordings of their lectures (Said et al., 2021). In Saudi Arabia, teachers of hard-of-hearing students used a special configuration of multiple spaces to enable the inclusion of synchronous sign-language translation in their online lectures (Alsadoon & Turkestani, 2020). In cases where the discrepancy between technology use by teachers and students was significant, teachers would often bridge the gap by adapting and adopting technologies (such as social media) that they were not always conversant with, but which were most used and preferred by students. As a teacher participant put it, teachers have ‘to go where [students] are, and not wait for [students] to come to where [they] are’ (Sales et al., 2020, p. 13).

Often teachers would consider the compatibility of certain technologies with their teaching philosophies and practices within their disciplines. Teacher educators in Israel, for example, might feel additional pressure from the expectation that their pedagogical use of technologies has to set examples for their student teachers (Hadar et al., 2021). As another example, teaching translation/interpretation in Mainland China was especially challenging during the ERT period since teachers have to demonstrate to students the operation of simultaneous interpretation equipment and the use of dual-track recording function—which is not commonly found in existing online applications (Ren, 2020).

#### Peer factors

Teachers reported that they saw their colleagues as not only sources of inspiration for technology use, but also remedies for stress and uncertainty during the ERT period (Ren, 2020). Unlike in prior ‘online teaching’ where they could still meet in person to discuss technology use, many teachers struggled with technological learning-by-doing in relative isolation during the COVID-19 lockdown period (Cutri et al., 2020). In view of the absence of physical spaces for colleagues to informally exchange professional practices and channel their emotionality and empathy for one another (Cutri et al., 2020; Scherer et al., 2021), some teachers put in deliberate effort into organising new networking spaces to bring the academic community together online. In an attempt to alleviate the uncertainties brought by ERT and their adverse impact on psychological well-being, teachers worked together remotely as a team to explore solutions and share useful insights about technology use in teaching. They felt empowered by the constant encouragement and motivational texts from their peers (Ren, 2020). Teachers thrived on establishing connections with technology-proficient colleagues whose technical expertise and guidance were relied upon (Bailey & Lee, 2020; Mouchantaf, 2020) and whose ingenious engagement with technologies inspired and were even assimilated into their own teaching practices. As a mitigation strategy to ease teachers’ hasty migration into ERT, mutual empowerment through facilitated discussions amongst colleagues meaningfully shaped the ways technologies were used by teachers in ERT.

#### Interplay of factors

Whilst we have delineated potential factors shaping technology use in ERT in a linear, point-by-point fashion, this list of non-exhaustive items should not be conceived as separate, stand-alone factors since they interact in a complex and nuanced way across various contexts. For instance, having little institutional support and no access to LMS or students’ information, some teachers in public HEIs in Egypt resorted to reaching students through popular social media. Teachers then explored on their own the ways in which they could continue teaching activities via these platforms which were new to them (Sobaih et al., 2020). As for teachers in an Israeli college, upon realising some Arabic female students refused to appear online due to their cultural values, they made allowance for students’ decisions to keep their cameras off (Hadar et al., 2021). But the inability to read students’ expressions during class added to the teaching challenges during ERT and demanded additional flexibility and pedagogical adjustments from teachers. Therefore, technology use is influenced by the combined factors of students’ socio-cultural backgrounds and teachers’ resources and adaptability to changes. In addition to the complex interplay of these factors, these examples demonstrate that teachers’ technology use in ERT is heavily contextualised across HE settings and should therefore be understood in its wider cultural embedding and socio-economic contexts.

### Implications of technology use in ERT for teachers

As for our second research question, the studies reviewed indicate that the implications of technology use in ERT for teachers are manifold. These findings are categorised into pedagogical, work-related, and cross-cutting implications, discussed below (see Table 9 for a summary table).

**Table 9:** Implications of technology use in COVID-19 emergency remote teaching for teachers as implicated in the reviewed studies

Category	Implications	Details/explanations	References
Pedagogical	Feeling of detachment from students	• Worsened classroom dynamics and more pronounced hierarchical teacher-student relationship in the new spatial-temporality	Cutri et al. (2020), Eringfeld (2021), Gyampoh et al. (2020), Hadar et al. (2021), Lu (2020), Marshalsey and Sclater (2020), Ren (2020)
		• Loss of informal spaces where students can interact further with teachers outside class	Cutri et al. (2020)
		• Gap between students’ and teachers’ uses of technologies	Callo and Yazon (2020), Sobaih et al. (2020)
	Feeling of the ‘intimacy of distance’	• Development of closer relationships with students (e.g., through learning more about students’ home environments)	Eringfeld (2021), Gao and Zhang (2020), Hadar et al. (2021), Kidd and Murray (2020), Zeng (2020)
		• Development of more care and empathy for students	Khoza and Mpungose (2020), Kidd and Murray (2020)
Work-related	Flexibility in time management	• Commuting time being freed up for student support and self-care	Eringfeld (2021), Kidd and Murray (2020), Tejedor et al. (2020)
	Work intensification	• Expectations and pressure from teachers themselves and others to work remotely for longer hours	Khan et al. (2020), Kidd and Murray (2020), Lu (2020), Marshalsey and Sclater (2020), Mouchantaf (2020), Said et al. (2021), Watermeyer et al. (2021)
		• Expanded teachers’ role and job functions to provide care and psychological support for students	Watermeyer et al. (2021)
		• Blurring home/workspaces, private/public boundaries	Khoza and Mpungose (2020), Kidd and Murray (2020), Watermeyer et al. (2021)
	Changing work relationships	• Maintenance of relationships with colleagues and organisation of spaces for peer commiseration and networking	Bailey and Lee (2020), Cutri et al. (2020), Khoza and Mpungose (2020), Mouchantaf (2020), Ren (2020), Scherer et al. (2021)
		• Less hierarchically-organised workplace for teachers	Eringfeld (2021), Tejedor et al. (2020), Watermeyer et al. (2021)
Cross-cutting	Undermining teachers’ work and the academic profession	• Teachers’ work being reduced to functions of a technician or a curator of digital resources	Watermeyer et al. (2021)
	Upholding ethics when teaching in the new context	• Recognition of the need to equip students with critical and reflective thinking capacity when studying and interacting with others online	Dampson et al. (2020), Ghounane (2020), Sales et al. (2020), Sobaih et al. (2020), Tejedor et al. (2020)
		• Teachers’ professional deliberation on the proper use of technologies in their teaching in the absence of a code of conduct	Cutri et al. (2020), Diningrat et al. (2020)

#### Pedagogical implications

With the paradoxical amalgam of being ‘together but (physically) apart’ (Marshalsey & Sclater, 2020) in the new COVID-19 context of teaching, the notions of space and time, as well as the dynamics of the classroom and teacher-student relationship, have undergone less palpable yet important changes.

Spatiality-wise, teachers realised the loss of important physical spaces and the erosion of values traditionally attached to these spaces during the transition to ERT. Marshalsey and Sclater (2020), for example, reason how a physical art and design studio embodies a distinctive set of values, resources, and the signature experiential hands-on pedagogical practice of their discipline. But when artworks are presented online, their materiality, colours, and texture may be diminished.

Temporality-wise, some teachers felt a strongly contorted notion of *time* which rendered futile any discussion on the ordinary longitudinal perception of ‘being ready for teaching’ (Cutri et al., 2020). Not only was the migration to ERT perceived as rushed and disorganised but teachers also felt time as short, discrete intervals when many changes could occur. Some even found it difficult to find ‘a point of reference for their sense of self as experienced professionals’ (Cutri et al., 2020, p. 533). This new sense of temporality is perhaps most concisely summarised by a comment made by a teacher during ERT: ‘I always plan a month ahead. Now I live from one day to the next’ (Hadar et al., 2021, p. 454).

Within this new spatial–temporal context, teachers often felt that student engagement in remote teaching and learning activities was superficial and unequally distributed (Joshi et al., 2020; Kidd & Murray, 2020). Deprived of in-person interaction, teachers can neither hear the voices nor see the expressions of all students, and find the classroom discourse to be dominated by students who are generally more confident in sharing their ideas in front of the whole class (Hadar et al., 2021; Marshalsey & Sclater, 2020). With the loss of informal physical spaces where students used to ask questions and interact further with teachers before and after class (Cutri et al., 2020), some teachers commented that both teachers and students were more likely to stay in their ‘echo chambers’ during the pandemic (Eringfeld, 2021).

Teachers adopted different strategies to navigate being outside the comfort zone of the physical classroom. Some attempted to retain or increase control over interactions in the remote ‘classroom’ (Mideros, 2020) such as by only letting students speak when allowed (Gyampoh et al., 2020) and shifting to a predominantly teacher-centric, didactic approach of lecturing because of the perceived difficulty of implementing hands-on training in an exclusively remote teaching environment (Cutri et al., 2020). The students, too, adopted their own strategies, often distinct from their teachers’ (Callo & Yazon, 2020; Sobaih et al., 2020). As some students generally adapted to ERT with relative ease (Mideros, 2020; Ren, 2020), sometimes they even used technology as a defensive wall to *exclude* teachers (who were in some cases less tech-savvy than their students) from being involved in their studies during the pandemic (Sales et al., 2020). Many teachers in the studies reviewed reported that the mandated use of various technologies in ERT puts a strain on pedagogy, the major implications of which may include an elevated feeling of detachment from the class, a heightened distance from students (Kidd & Murray, 2020), and a more pronounced gap in teacher-student interactions (Callo & Yazon, 2020; Sales et al., 2020).

Moreover, ERT is thought to have precipitated the collapse of ‘yishigan’ (仪式感)—a Chinese expression which, when applied to this context, refers to the sense that teaching is a special, ritualised occasion (Lu, 2020; Ren, 2020). As ‘yishigan’ abates in the context of ERT, so does the sense of formality and immediacy felt by teachers and students, both of whom may no longer view teaching and learning as a serious, formalised routine of life in the same way as before; some of the studies reviewed note that motivation and classroom engagement are lowered as a result of this change in perception (see examples in Joshi et al., 2020; Lu, 2020; Marshalsey & Sclater, 2020).

In contrast with the sense of limitation, hierarchy, and loss illustrated by the accounts summarised above, other teachers reported a sense of the ‘intimacy of distance’ and a less visible teacher-student hierarchy as a combined result of emergency technology use during the pandemic. Such teachers valued the creation of spaces for more student-oriented and student-empowering pedagogy. In Mainland China, for example, the classroom atmosphere was livened up as students were encouraged by teachers to engage in class via alternative forms of interaction online such as sending emojis, raising ‘hands’, and taking polls (Gao & Zhang, 2020; Zeng, 2020). In other contexts, teachers felt an idiosyncratic sense of closeness as they shared a screen and read the same text with students on their devices (Eringfeld, 2021). They also reported a better understanding of students’ personal circumstances, home environment, and even household responsibilities as students turned on their cameras in class (Hadar et al., 2021; Kidd & Murray, 2020). In many ways, teachers observed their students being more relaxed in class, which enabled teachers to build personal relationships with their students in ways that they had never envisioned before (Marshalsey & Sclater, 2020).

Because of the collapse of ‘yishigan’ and the resultant casual and more relaxed classroom dynamics in the new spatiality, some teachers adapt to the ‘online etiquette’ by using emojis and GIFs when communicating with students (Marshalsey & Sclater, 2020). Also, the fact that students may be more technology-competent than teachers meaningfully shifts the dynamic of the teacher-student relationship in the ERT classroom (Kidd & Murray, 2020), for teachers often solicited help from students on questions regarding technology use, and during this process teachers increasingly saw students as their partners in teaching rather than subordinates to themselves (Cutri et al., 2020). As Cutri et al. (2020) remark, ‘the negative connotations of risk-taking and making mistakes while learning to teach online seem to have been mitigated by a combination of affective factors such as humility, empathy, and even optimism’ (p. 523). As an experience of vulnerability, ERT has grounded and humbled teachers, allowing them to develop both more appreciation for self-care (Eringfeld, 2021), and more empathy for students (Khoza & Mpungose, 2020; Kidd & Murray, 2020).

Teachers realised the salience of exercising care for students and themselves and considering the emotionality of students, especially those in vulnerable states (Alqabbani et al., 2020; Sales et al., 2020). Pastoral care took priority during particularly distressing periods when students were most in need of emotional support (Sobaih et al., 2020; Tejedor et al., 2020). All these examples suggest that under the new spatial–temporal reorientation an intricate web of human relations has evolved and, to varying degrees, been revitalised.

#### Work-related implications

The task of transitioning teaching to an alternative mode is only one of the many challenges teachers face in the larger contexts of academia during the pandemic period (Cutri et al., 2020). Although the extra time seemingly freed up by, say, the lack of commutes is highly valued for student support, self-care or family care (Eringfeld, 2021; Kidd & Murray, 2020; Tejedor et al., 2020), there has also been an excessive intensification of workload in preparation for ERT (Khan et al., 2020; Lu, 2020; Mouchantaf, 2020; Said et al., 2021), and this is expected to last for a few years into the post-ERT era (Watermeyer et al., 2021). When working from home, teachers received as many as hundreds of students’ inquiries throughout the day via various applications (Alsadoon & Turkestani, 2020; Sobaih et al., 2020). Coupled with the pressure to prove that work has been conducted remotely (Kidd & Murray, 2020; Marshalsey & Sclater, 2020), some teachers report feeling compelled to be present online around the clock. The ‘timelessness’ of working remotely in a home setting has been succinctly summarised by a teacher: ‘it is too easy to “just send one more email”’ (Watermeyer et al., 2021). The praxis and boundaries of academic work were shifted and reconstructed in ways many perceived as intrusive into the personal life sphere and deteriorative to work-life balance and also teachers’ well-being and occupational welfare (Watermeyer et al., 2021).

In addition, with looming financial challenges to the HE sector, casualised and untenured staff reported an elevated feeling of job precarity because their extra commitment to teaching cuts into time for other academic work, such as publishing research—which they perceived as often prioritised over teaching efforts in HE career progression (Cutri et al., 2020). Some reported that these teachers’ vulnerability was compounded by the management’s misperception that teaching remotely during emergency lightens teachers’ workload, and by their misinterpretation that low scores given by students on evaluations of ERT are a marker of ‘teacher quality’ rather than a way for students to express disinclination towards ERT in general (Watermeyer et al., 2021).

Technology use in ERT was further complicated by the need for swift re-coordination of private routines and domestic spaces to make room for professional work. A teacher, for example, asked all household members to disconnect from the Wi-Fi when teaching (Kidd & Murray, 2020). Having a separate, free-of-disturbance workspace at home is a luxury that not many teachers could afford (Gyampoh et al., 2020; Joshi et al., 2020) especially in contexts like Pakistan where joint families may live together in a crowded household (Said et al., 2021). Due to the non-separation of home/workspaces, customary parameters between the private and public domains were being reconstituted, and the boundaries between teachers’ personal and professional identities became blurry (Khoza & Mpungose, 2020). Consequently, female academics with caring responsibilities were disproportionately affected, and increasingly teachers found themselves struggling to perform either role well (Watermeyer et al., 2021).

In the larger context of HE, teachers were also worried about the ‘placelessness’ of HE during lockdowns and that the role of HE as an embodied, communal space for teaching and learning, self-formation, and socialisation was being undermined (Eringfeld, 2021). In two studies based in the UK (Eringfeld, 2021; Watermeyer et al., 2021), the accounts of their teacher participants add up to a strong ‘dystopian’ rhetoric, reflecting their fears that the ERT migration epitomises the beginning of a prolonged contraction of HE as an on-campus experience and monetisation of part of the HE experience driven largely by massification but not quality, thereby undermining the core academic values and humanising aims of HE.

Not all studies reviewed painted a consistently gloomy picture of the work-related implications of ERT and technology use. Some studies note that the compulsory, emergency move to remote teaching may have offered multiple opportunities. For example, in some propitious circumstances, teachers were able to constitute their networking spaces online to channel mutual support and facilitate exchanges on technology use. There are also reports that more trust was placed on technology specialists, technicians, and younger faculty who were often seen as more technologically adept and relied upon during ERT (Watermeyer et al., 2021). Moreover, the infrastructural divisions that used to separate departments on a physical campus are largely dismantled with the migration to ERT, enabling possibilities of various forms of inter-departmental communication and cross-disciplinary collaboration (Tejedor et al., 2020) and thereby making HE a flatter-structured and less hierarchically-organised workplace for teachers (Eringfeld, 2021).

#### Cross-cutting implications

Some of the teachers in the studies reviewed commented on the potential of ERT to undermine the ethos of the academic profession and imperil the work of academics. They noted that ERT could be pedagogically regressive, as teachers’ role may be reduced to merely technical functions, such as uploading materials online. This challenged their beliefs about what good teaching entails and compromised their often long-established pedagogical practices (Watermeyer et al., 2021). Other teachers struggled with balancing depth in their teaching with what they saw as their students’ preference for over-simplified yet visually appealing inputs such as bite-sized explanations shared on TikTok and other social media (Sales et al., 2020). Some anticipate worrying trends of ‘dumbing down’ of HE if teaching continues to be impersonal, disembodied and mediated predominantly by digital technologies in the post-ERT era (Watermeyer et al., 2021).

We have discussed so far the changes to HE teaching due to the relocation to newly formed spaces, as reported in the studies reviewed. Yet, some principles and values that teachers apply to guide their teaching practices remained unchanged amidst the ongoing crisis. These include the upholding of integrity, academic transparency, privacy, and other ethical principles in teaching (Mouchantaf, 2020). For example, teachers were concerned about the potential collection of students’ data for third-party use without prior informed consent (Diningrat et al., 2020; Joshi et al., 2020). Others also recognise the importance for students of using technology responsibly (Gyampoh et al., 2020) and being equipped with critical and reflective thinking capacity to evaluate the accuracy and relevance of information online (Sales et al., 2020; Tejedor et al., 2020), including resisting the temptation to reuse others’ ideas as their own work (Dampson et al., 2020) and refraining from using improper language on social media (Ghounane, 2020; Sobaih et al., 2020). This was especially relevant during the absence of teacher’s in-person monitoring, when the responsibility to access and study educational materials was partially shifted to students (Gyampoh et al., 2020), many of whom were inclined to explore topics of interest on their own (Marshalsey & Sclater, 2020; Mideros, 2020; Sales et al., 2020).

For teachers themselves, their practical wisdom and professional deliberation to ‘consider when, why, and how to use technology properly’ (Diningrat et al., 2020, p. 706) were put to the test during the emergency contexts of teaching. A teacher participant in the study by Cutri et al. (2020) shared his belated reflection on an inadvertent, frivolous ridicule he had made about a student’s slow internet speed in front of the entire class online. This anecdote alludes to two problems looming in the wider context of HE teaching: (1) the largely absent code of conduct that delineates appropriate practices and roles of teachers and students in the new spatiality (and this can be due partly to the short time horizon in ERT); and (2) the difficulty for teachers to create supportive yet private spaces to address equity issues and attend to students’ emotionality in strict confidence when being online (Cutri et al., 2020).

## Conclusion

Teachers participating in the studies reviewed in this paper indicated a multiplicity of factors that interacted to shape their technology use during the ERT period. In line with Liu et al. (2020)’s pre-pandemic work, we find strong evidence that technology use in teaching is a context-sensitive, socially-embedded topic of study and hence should be understood in the socio-political, cultural and material context in which academics and students are situated (Selwyn et al., 2020). For example, the label ‘technical issues’ could encompass a wide range of contextualised problems, from power outages to long commutes for Internet access, from material shortages to widespread hunger, from trenchant poverty to deep-seated structured inequalities, which afflict disproportionately relatively poor, underserved communities and the most disadvantaged segments of populations (Chan et al., 2022) but are also palpable within higher-income countries/regions [see, for example, Cullinan et al. (2021) for a study on broadband access disparities in Ireland].

The narrative account we constructed is indicative of the resourcefulness and resilience of teachers to continue teaching during the crisis, even those in marginalised communities where resources are limited. This view is also shared by Padilla Rodríguez et al. (2021) who study the changes teachers in rural Mexico have made to their teaching practice in response to the suspension of in-person classes without receiving much external support during the pandemic. Around the world, teachers forayed into ERT during times of uncertainty by seeking to empower themselves and exploring various technological artefacts in teaching on their own, on the one hand; and by endorsing mutual empowerment and drawing inspiration from amongst their peers, on the other. Their collective efforts in supporting one another in the wake of crisis created what Matthewman and Uekusa (2021) call ‘disaster communitas’, which temporarily served to support teachers when adapting to the hasty conversion to ERT. We concur with Hickling et al. (2021) that the creation of a supportive space and environment for HE teachers to commiserate, discuss experiences, and share insights and resources with colleagues helps advance teaching practices with technology.

In answering the second research question, we have discussed at length the implications of a more encompassing use of technology in ERT and how evolving notions of space and time combined to reconstitute teacher-student relationships and the nature of academics’ work (Williamson et al., 2020). The studies reviewed indicate that the rushed transition to ERT has affected the sense of professional identity of academics as HE teachers (Littlejohn et al., 2021) in ways that are as yet only partly explored. Echoing the findings of Ramlo (2021), we believe that teachers’ negotiation of the blurring home-workspace boundaries (Blumsztajn et al., 2022; Littlejohn et al., 2021) and attempts to rebalance their professional work and personal life have important implications for future HE teaching and merit further investigation (Gourlay et al., 2021).

As COVID-19 continues to take a toll on people’s lives, we draw on the studies reviewed to emphasise the importance of re-prioritising the value of social and emotional connections in HE teaching, as well as the overall well-being of both teachers and students (Baker et al., 2022; Yeung & Yau, 2021). ‘Networks of care’ between teachers and students as well as amongst teachers themselves may be constructed to ameliorate uncertainties brought by the pandemic (Czerniewicz et al., 2020; Joseph & Trinick, 2021). Elements of care can be developed by simple acts of kindness (Murray et al., 2020) and gestures to communicate approachability (Glantz et al., 2021), all of which contribute to constructing more supportive and less hierarchical teacher-student relationships in the digital context. We note, however, that evidence scattered across the studies reviewed indicates that academic recognition and reward systems have not accounted well for the creative efforts that academics (including casualised and untenured staff) have put into teaching and maintaining relationships with their colleagues and students in response to the ongoing challenges ensuing from the coronavirus crisis. This is another priority for HEIs and leadership teams. On a final note, future research may explore further, innovative ways in which HE teaching can be reconstituted in the presence and context of technology without undermining teachers’ professional identity or compromising the revitalisation of teaching as an embodied, communal, and humanising experience as campuses around the world re-open, in full or in part, for in-person activities in post-pandemic times.

## Appendix

### Appendix 1. A detailed version of inclusion/exclusion criteria

**Table:** 

	Inclusion	Exclusion
Publication types	Peer-reviewed original empirical research journal articles	Books, reviews, opinion and reflection pieces, conference proceedings, and non-peer-reviewed articles
Publication date	Published in 2020 (including those published ahead of print in 2020)	Not published in 2020
Languages	Written in English and/or in Chinese	Written in other languages than in English or Chinese
Focus of study	Focus on technology use in emergency remote teachingT from teachers’ perspectives	Focus on technology use in non-teaching domains or emphasise other stakeholders’ perspectives
Settings	Data collected during and/or after the COVID-19 outbreak in higher education settings, i.e., Levels 6 to 8 of the International Standard Classification of Education 2011 (UNESCO Institute for Statistics, 2012)	Data collected before the COVID-19 outbreak and/or in non-higher education settings
Disciplinary areas	At least 50% of higher education teacher participants are from humanities, arts, and social sciences (HASS) disciplines, which can be readily mapped against the Common Aggregation Hierarchy disciplinary groupings 14 to 23 in *Higher Education Classification of Subjects* (Higher Education Statistical Agency, n.d.)	Over 50% of higher education teacher participants are from science, technology, engineering, maths, medicine (STEMM), and other non-HASS disciplines

### Appendix 2. Search terms in English and Chinese (note that the search strategy varied slightly across databases due to the different limits they set on the length of search input)

**Table:** 

Key terms	Higher education		Technology-related		Teaching		COVID
Version 1 (Dimensions.ai, EBSCO, SAGE, ProQuest, Scopus, Web of Science)	("higher education" OR tertiary OR universit* OR college* OR post-secondary OR "post secondary" OR postsecondary OR faculty OR professor* OR lecturer*)	AND	(online OR on-line OR e-learn* OR elearn* OR remote* OR virtual* OR "virtual reality" OR "augmented reality" OR “mixed reality” OR distance educat* OR distance teach* OR distance learn* OR digital* OR learning platform* OR technolog* OR ICT OR instruction* technolog* OR education* technolog* OR edtech OR learning platform* OR learning technolog* OR technology-enhanced OR telecommunicat* OR tele-communicat* OR tele-conferenc* OR teleconferenc* OR multimedia OR "multi media" OR multi-media OR web* OR learning site* OR cyberlearning OR video* OR Zoom OR mobile app* OR "mobile learning" OR m-learn* OR mlearn* OR mobile technolog* OR LMS* OR Learning Management System* OR "social media" OR social network* OR SNS* OR facebook OR twitter OR instagram OR youtube OR whatsapp OR MOOC* OR massive open online course* OR OER OR Open Educational Resource* OR synchronous OR asynchronous OR flexible learn* OR blended learn* OR hybrid learn* OR flipped class* OR game* OR gamif* OR collaborat* platform* OR forum* OR e-forum* OR online forum* OR blog* OR portfolio* OR Google OR "artificial intelligence" OR AI)	AND	(teach* OR educat* OR instruct* OR pedagog*)	AND	(COVID OR COVID-19 OR coronavirus OR CoV OR CV-19 OR SARS-CoV-2 OR 2019-nCoV OR pandemic*)
Version 2 (ACM, PsychINFO, WHO)	Same as above	AND	(online OR on-line OR e-learn* OR remote* OR virtual* OR distanc* OR digital* OR digiti* OR technolog* OR edtech OR media OR web* OR synchronous OR hybrid OR blended)	AND	Same as above	AND	Same as above
Version 3 (IEEE Xplore, Google Scholar)	(“Higher Education” OR University OR Faculty)	AND	(Online OR Education* Technolog* OR Digital* OR Virtual* OR E-learning)	AND	same as above	AND	(COVID-19 OR coronavirus OR pandemic)
Chinese databases (CNKI, CQVIP, Wanfang)	(大学 + 高等教育 + 学院 + 高等学校 + 高校 + 院校 + 本科 + 研究生)	AND	(线上 + 在线 + 网 + 远程 + 远距离 + 遥距 + 云端 + 视频 + 科技 + 平台 + 电子 + 百度 + 微博 + 抖音 + 慕课 + 直播 + 雨课堂 + 钉钉 + 微信 + QQ + 腾讯 + "Zoom" + 超星)	AND	(课堂 + 教师 + 教室 + 課程 + 教育 + 老师 + 讲师 + 教授 + 学生 + 学习 + 学堂 + 教学)	AND	(COVID + COVID-19 + coronavirus + corona + 新型冠状 + 新冠 + 病毒 + 肺炎 + 疫情 + 停课)

### Appendix 3. PRISMA 2020 flow diagram for systematic review (Page et al., 2021)


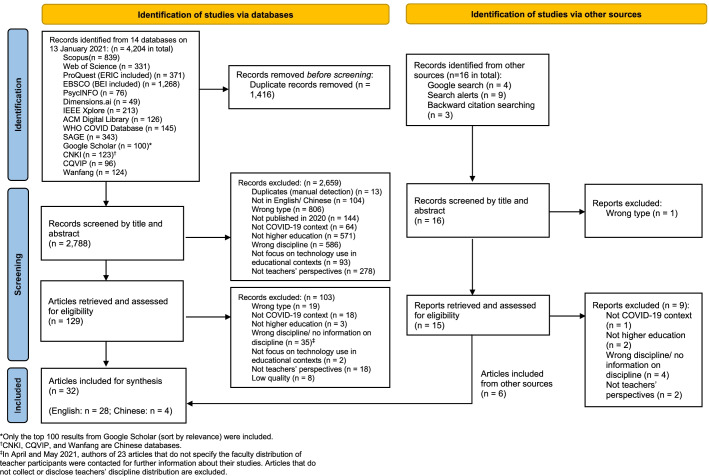


### Appendix 4. Quality and relevance assessment rubric and the average scores of the 32 included studies (adapted from Oancea et al., 2021)

**Table:** 

Assessment criteria	Strength of conceptualisation or theory	Rigour in argument and empirical study	Appropriateness of approach	Well-grounded conclusions and recommendations	Thoughtful discussion and interpretation	Relevance to this systematic review
Explanation	• Critical engagement with the concepts • Critical use of terminology	• Detailed, critical presentation of the warrant for the research • Strong, error-free design • Awareness of limitations	• Methods and analysis fit RQ(s) and study objective(s) • Consistency of focus • Alignment of analytic techniques and data collection	• Conclusions and recommendations clearly arising from evidence and argument presented • Appropriate and warranted generalisations	• Richness of insight, including (potentially unique) understanding of the field • Appropriate depth, reflection, and criticality	• Coverage and foci of study overlap extensively with those of this review
Average score of studies included (out of 4.0)^a^	2.38	3.0	2.91	2.81	2.91	2.97

^a^Score description: 4—criterion fully met; 3—criterion mostly met, though with some weaknesses; 2—criterion only partly met, with several or serious weaknesses; 1—criterion largely not met

### Appendix 5. Data extraction grid

**Table:** 

No	Items to extract	Description	Reviewers’ column
1	Reference	• Include the reference of paper using the APA in-text citation style	
2	Authors’ affiliation(s)	• If more than one author, state the first author's affiliation first	
3	Funder	• State all source(s) of funding	
4	Focus of study	• State all major research foci, topics, and objectives	
5	RQ(s) or hypotheses	• State all RQ(s), problem statement(s) and/or hypothes(es)	
6	Target population	• State the target population of the study • Include details of the HE institutions under study • Name the countries/regions that the institution(s) under study are in	
7	Theoretical underpinnings	• State all theories or models used to support research formulation and analysis	
8	Conceptualization of technology	• Discuss how the concept of ‘technology’ and terms alluding to it are defined, used, and conceptualized throughout the paper	
9	Conceptualization of ‘emergency remote teaching’	• Discuss how the concept of ‘emergency remote teaching’ and terms alluding to it are understood (often in relation to regular ‘online teaching’) throughout the paper	
10	Methodology	• State the details of research approach, methods used, and rationale (if any) for such methodology	
11	Sampling	• Include details such as population size, sampling strategies, sampling frame, and sample size	
12	Data collection and recruitment	• Include participant recruitment strategies, response rates, and other information (including pilot studies) about collecting data from participants	
13	Context of study	• Include details such as the duration of data collection, the country/region’s COVID-19 infection rates and government reactions, HE management policies and arrangements during the period of study	
14	Teacher participants’ characteristics	• Include details e.g. age, gender, educational attainment, years of experience, academic rank, employment status, disciplines, and any other demographic and descriptive information about HE teacher participants	
15	Data analysis	• Include the analytical approaches and methods used by researcher(s) to analyse their data collected from participants	
16	Findings	• Highlight all major findings, implications, results, and conclusions of the study	
17	Limitations	• Include the study limitations (if any) and measures to overcome these limitations (if any)	
18	Suggestions	• Include the suggestions for future research and/or practice	
19	Other	• Include other details e.g. research ethics and researchers’ positionality • Discuss anything else of interest yet uncaptured by the above categories	

### Appendix 6. Summary of characteristics of 32 reviewed studies

**Table:** 

References^a^	Country	Remit	Discipline	Participants (at HE level)	Teacher sample	Approaches	Main focus (in relation to HE teachers in the context of COVID-19 ERT)
Akyürek (2020)	Turkey	National	Music	Teachers	46	Mixed (interview)	Teachers’ preparation, planning for ERT and problems faced
Alqabbani et al. (2020)	Saudi Arabia	Local	Multi-discipline	Teachers	401	Quantitative (survey)	Teachers’ readiness, perceived effectiveness and attitudes towards ERT
Alsadoon and Turkestani (2020)	Saudi Arabia	Local	Multi-discipline	Teachers	11	Qualitative (interview)	Obstacles teachers of hearing-impaired students faced during ERT
Bailey and Lee (2020)	South Korea	National	Language	Teachers	43	Quantitative (survey)	Expected benefits and challenges of implementing ERT for teachers of different online teaching experiences
Callo and Yazon (2020)	The Philippines	Local	Multi-discipline	Students and teachers	348	Quantitative (survey)	Factors influencing teachers’ readiness for ERT
Cutri et al. (2020)	United States	Local	Education	Teachers	30	Mixed (survey and interview)	Teachers’ readiness for ERT, especially the affective and cultural dimensions
Dampson et al. (2020)	Ghana	Local	Education	Students and teachers	20	Mixed (survey and interview)	Teachers’ perceived SWOT of using their university’s Learning Management System
Diningrat et al. (2020)	Indonesia	National	Education	Teachers	73	Quantitative (survey)	Teachers’ perceived barriers to ERT and general pedagogical competencies
Eringfeld (2021)	United Kingdom	Local	Education	Students and teachers	4	Qualitative (interview and podcasting for sound elicitation)	Teachers’ utopian hopes and dystopian imaginaries for higher education during and after the pandemic
Gao and Zhang (2020)	China	Local	Language	Teachers	3	Qualitative (interview and written reflections)	Teachers’ cognitions about ERT and acquisition of ICT literacy at the initial outbreak of COVID-19
Ghounane (2020)	Algeria	Local	Language	Students and teachers	8	Mixed (survey and interview)	Teachers’ motivations and views of using Moodle and social media in ERT
Gyampoh et al. (2020)	Ghana	Provincial	Education	Teachers	24	Qualitative (interview)	Teachers’ perceived personal and institutional readiness for ERT
Hadar et al. (2021)	Israel	Local	Education	Teachers	33	Qualitative (survey and interview)	Adaptation of teaching methods in the clinical component of teacher education preservice curriculum and the shift to social emotional learning during ERT
Joshi et al. (2020)	India	Provincial	Multi-discipline	Teachers	19	Qualitative (interview)	Barriers faced by teachers when conducting ERT in different home settings
Khan et al. (2020)	Bangladesh	National	Language	Teachers	22	Qualitative (interview)	Challenges faced by teachers in ERT and teachers’ suggestions for overcoming them
Khoza and Mpungose (2020)	South Africa	Local	Education	Teachers	20	Qualitative (survey and interview)	Teachers’ transformation experiences and values that facilitated the embracing of the ‘digitalised curriculum’ during ERT
Kidd and Murray (2020)	United Kingdom	Provincial	Education	Teachers	14	Qualitative (survey and interview)	Teachers’ experiences and challenges in the ERT period of moving the preservice teacher education practicum to new online spaces
Lu (2020)	China	Local	Interpretation	Students and teachers	10	Mixed (survey and interview)	Comparison between students and teachers’ experiences, perceived effectiveness, benefits, and shortcomings of ERT
Marshalsey and Sclater (2020)	Australia	Local	Art & design	Students and teachers	9	Qualitative (survey and secondary data)	Teachers’ involvement with online tools and platforms and their lived experiences during ERT
Mideros (2020)	Trinidad and Tobago	Local	Language	Students and teachers	8	Qualitative (survey and interview)	Teachers’ attempts to promote out-of-class learning of Spanish during the period of ERT
Mouchantaf (2020)	Lebanon	National	Language	Teachers and administrators	50	Quantitative (survey)	Factors affecting the smooth transition to ERT and teachers’ perceived advantages and disadvantages of ERT
Ren (2020)	China	Local	Interpretation	Students and teachers	31	Mixed (survey and social media analysis)	Teachers’ experiences, communications with colleagues, and changes in attitudes and competencies during ERT
Said et al. (2021)	Pakistan	Local	Business	Teachers	7	Qualitative (interview)	Teachers’ lived experiences, attitudes, and challenges during ERT
Sales et al. (2020)	Spain	National	Multi-discipline	Teachers	20	Qualitative (interview)	Teachers’ attitudes towards ERT and perceptions of students and their own levels of ‘information and digital competence’
Scherer et al. (2021)	58 countries worldwide	Global	Multi-discipline	Teachers	739	Quantitative (survey)	Factors associated with the profiles of different teachers’ readiness for ERT
Sobaih et al. (2020)	Egypt	National	Tourism and hospitality	Students and faculty	304	Mixed (survey and interview)	Comparison of students and teachers’ uses of social media and challenges faced by them
Tang et al. (2020)	China	Local	Multi-discipline	Teachers	331	Quantitative (survey)	Teachers’ attitudes towards ERT and their prior experiences in online teaching
Tanga et al. (2020)	South Africa	Provincial	Social work	Students and teachers	12	Qualitative (interview)	Teachers and students’ experiences, attitudes, and challenges when implementing ERT
Tartavulea et al. (2020)	13 European countries	Regional (Europe)	Economics and business	Students and teachers	114	Quantitative (survey)	Teachers’ use of technologies in ERT compared to before, factors influencing the ERT process, the impact and effectiveness of ERT
Tejedor et al. (2020)	Spain, Italy, Ecuador	Multi-national	Multi-discipline	Students and teachers	196	Quantitative (survey)	Teachers’ attitudes and their perceived positive and negative aspects of ERT
Watermeyer et al. (2021)	United Kingdom	National	Multi-discipline	Teachers	1,148	Mixed (survey)	Teachers’ feelings and experiences with ERT, and the impact of it on teachers’ role, their work, and the higher education sector
Zeng (2020)	China	Provincial	Multi-discipline	Students and teachers	627	Quantitative (survey)	Teachers’ pre-COVID experience in online teaching and the impact of ERT on teachers’ work

^a^The references of four articles show the publication year of 2021. These four articles were published online ahead of print in 2020 and hence are included in this study

## Data Availability

All data generated or analysed during this study are included in this published article.
